# A fully automated pipeline for quantitative genotype calling from next generation sequencing data in autopolyploids

**DOI:** 10.1186/s12859-018-2433-6

**Published:** 2018-11-01

**Authors:** Guilherme S. Pereira, Antonio Augusto F. Garcia, Gabriel R. A. Margarido

**Affiliations:** 1 University of São Paulo, “Luiz de Queiroz” College of Agriculture, Department of Genetics, Av Pádua Dias, 11, Piracicaba, 13400-970 Brazil; 2 Present Address: North Carolina State University, Bioinformatics Research Center, 1 Lampe Dr, Raleigh, Campus Box 7566, 27607 USA

**Keywords:** Genotyping-by-sequencing, Ploidy estimation, Allele dosage, Population structure, Linkage mapping, GWAS

## Abstract

**Background:**

Genotyping-by-sequencing (GBS) has been used broadly in genetic studies for several species, especially those with agricultural importance. However, its use is still limited in autopolyploid species because genotype calling software generally fails to properly distinguish heterozygous classes based on allele dosage.

**Results:**

VCF2SM is a Python script that integrates sequencing depth information of polymorphisms in variant call format (VCF) files and SuperMASSA software for quantitative genotype calling. VCFs can be obtained from any variant discovery software that outputs exact allele sequencing depth, such as a modified version of the Tassel-GBS pipeline provided here. VCF2SM was successfully applied in analyzing GBS data from diverse panels (alfalfa and potato) and full-sib mapping populations (alfalfa and switchgrass) of polyploid species.

**Conclusions:**

We demonstrate that our approach can help plant geneticists working with autopolyploid species to advance their studies by distinguishing allele dosage from GBS data.

**Electronic supplementary material:**

The online version of this article (10.1186/s12859-018-2433-6) contains supplementary material, which is available to authorized users.

## Background

Genotyping-by-sequencing (GBS) has been applied to several genetic studies in a range of species (see [1–3]) for discovering variants, such as single-nucleotide polymorphisms (SNPs) and insertion-deletion (indels), at a relatively low-cost and with no prior genomic information [4]. It has proven to be very useful for agriculturally important plant species because, while genomic resources may be scarce, short reads from next generation sequencing (NGS) technologies can still be obtained. Standard GBS protocols, generally based on [5], rely on reduced genome representation libraries generated by restriction enzymes. In the fragment ends, barcode adapters are linked for sample multiplexing. Besides limiting the regions to be sequenced (e.g., methylation-sensitive enzymes potentially avoid repetitive regions), the restriction enzyme also influences the read depth (e.g., 6-bp rare cutters result in fewer regions to compete for amplification and sequencing reagents). In addition, read counts may be increased by sequencing the same library more than once, by reducing the multiplexing level or by size selecting DNA fragments to be sequenced. In general, genotype calling is based on a binomial likelihood ratio method that leverages read depth information, as implemented in pipelines such as TASSEL-GBS [6]. Finally, genotype calls and read depths are stored in variant call format (VCF) files.

For inbred diploid species, the read depth required for accurate genotype calling is rather low, because only homozygotes (let us say, AA and CC) have to be distinguished and thus common GBS practices (i.e., more frequent cutters, single sequencing run, and even a 384-plex library) are expected to perform well, especially if imputation is facilitated by the availability of a reference genome [7]. On the other hand, for hybrids and outbred species, the correct identification of heterozygotes (e.g., AC) becomes trickier when using very limited read depths. The challenge for effective use of GBS in autopolyploid species is even larger, because of the requirement to distinguish between more than one class of heterozygotes. In an autotetraploid biallelic locus where A and C are the respective reference and alternative alleles, for instance, apart from the homozygotes, AAAA (nulliplex) and CCCC (tetraplex), we might expect three different classes of heterozygotes: AAAC (simplex), AACC (duplex) and ACCC (triplex). Therefore, as the ploidy level increases, it becomes increasingly difficult to distinguish heterozygotes.

The correct allele dosage classification should greatly enhance genetic studies for these polyploid species. Biparental crosses involving nulliplex-simplex loci generate only two genotypes segregating in a progeny in a 1:1 ratio. This has allowed for linkage mapping studies using the double-pseudo testcross approach [8] of obtaining two separate, parental maps. Although widely used, this strategy generates a biased view of the recombination events among the progeny. This approach also limits the use of higher dosage loci [9]. A better strategy would be to build integrated maps (e.g., [10]), using additional segregation ratios (i.e., 3:1, 1:2:1 and 1:1:1:1) based on currently available methodologies [11–13] and tools [14, 15]. Despite the limitation for polyploids, these approaches have been successfully used to map single dosage markers (SDMs) segregating in 1:1 and 3:1 ratios in sugarcane (e.g., [16, 17]). Ideally, linkage mapping analysis for polyploids should take multiple dosage markers (MDMs) into account. Properly modeling allele dosage instead of using diploid-like genotypes could provide improved association power and prediction accuracies to genome-wide association studies and genome-based prediction.

Quantitative genotype calls can be achieved by using any SNP-based technique that provides a preferably unbiased measure of each allele amount, such as chip arrays or mass spectometry-based technology (see [18] for details). However, these technologies generally rely on solid, well-established genomic resources, such as reference genomes, which may remain inaccessible for many non-model species in a long term. Statistical models have been implemented in an attempt to distinguish different allele dosage classes based on the relative proportion or ratio between two alleles. Although some tools are currently available [19, 20], only SUPERMASSA [21] addresses the dosage calling problem through genetic models of expected class frequencies within a Bayesian network. In addition to a classification model without any genetic assumptions, it can use genetic models considering either F_1_ expected segregation or Hardy-Weinberg Equilibrium (HWE) allele frequencies to assign individuals into dosage classes. More importantly, it allows for the estimation of the most likely ploidy level when it is unknown or varies along the genome or among individuals [21]. This approach was validated for sugarcane, a complex polyploid [9].

Despite these advantages, SUPERMASSA was not previously available as a user-friendly tool for the analysis of thousands of variants in the standard VCF format. The method was originally designed for data generated with the Sequenom iPLEX MassARRAY^®;^ platform [22], which often yields small numbers of markers that can be manually analyzed. It is thus still largely inaccessible to the majority of bioinformatics end users, hampering widespread practical application of high throughput genotyping of polyploids.

In this context, the software presented here, VCF2SM, aims to integrate the use of polymorphic loci detected by sequencing approaches and the SUPERMASSA software for quantitative genotype calling. A modified TASSEL-GBS software for obtaining VCF files with exact read depths from GBS is also provided. This is necessary because the software cannot deal with the high read depths required for polyploids. Publicly available GBS experiments in diverse populations from two autotetraploid species, potato (*Solanum tuberosum* L.) [23] and alfalfa (*Medicago sativa* L.) [24], were used to test the software. In addition, F_1_ mapping populations of alfalfa [25] and switchgrass (*Panicum virgatum* L.) [26], a tetraploid species with diploid behavior, were used for inferring putative segregation at higher dosage loci. These datasets were particularly important because they contain both diverse panels and mapping populations, such that further structure and linkage analyses could also be performed.

## Results and discussion

### The TASSEL-GBS pipeline modified for polyploids

Earlier implementations of TASSEL-GBS (v.3 and 4) output truncated read counts per allele in the VCF file. The most recent version (v.5) of the pipeline addressed this limitation, but provides only approximate values for higher read counts [7]. The read counts are then used for genotype calling [6], which works fine for diploid calls. However, in polyploids, GBS pipelines optimized for increasing the read depth often generate much higher read counts. The ratio of read counts is used to inform on the proportion between the two alleles and, ultimately, on the dosage. Thus the current approximation provided should be avoided for quantitative genotyping purposes.

Here, we modified the TASSEL4 (v.4.3.7) software, whose GBS pipeline originally returned depths of up to 127. Now, the so-called TASSEL4-POLY has increased the limit to 32,767, in order to get their exact counts. For running this modified TASSEL-GBS pipeline, one should change the flag -y to -sh when using the FastqToTBT and DiscoverySNPCaller plugins. One of the main consequences of modifying the pipeline for storing larger read depths is a higher memory requirement. Roughly, each TASSEL-GBS plugin that uses the -sh flag requires twice as much memory as the original version. TASSEL4-POLY can be downloaded at https://github.com/gramarga/tassel4-poly. Alternative software can be used to identify polymorphisms and generate VCF files, such as FREEBAYES [19] and GATK [27], as long as they provide allele depth counts.

### The VCF2SM pipeline

VCF2SM was written in Python and consists of a single command-line function. Users can run it directly from their operating system prompt. Its arguments include both SUPERMASSA and VCF2SM options (please see https://github.com/gramarga/vcf2sm for argument details). It takes a VCF file containing exact read depths, as input, and outputs a VCF file with polyploid genotype calls, i.e., depicting reference and alternative allele dosages. For instance, if an autotetraploid individual is ACCC, its output genotype will be 0/1/1/1, indicating single dose for the reference allele A and triple dose for the alternative allele C.

The path for input files should be provided after the –input or -i flag, whereas output files are given by –output or -o. The same + (plus sign) from TASSEL-GBS can be used as a wildcard for file (usually, chromosome) number, with starting and ending numbers indicated by –sF and –eF, respectively. The path for the SUPERMASSA script is required and should be indicated through –SMscript. Additional flags indicate several native filtering arguments as well as the values for running SUPERMASSA, as described next. Python implementation of SUPERMASSA is available at https://bitbucket.org/orserang/supermassa.

#### SUPERMASSA options

The SUPERMASSA software can implement three different inference models. The type of inference is designed by the –inference or -I flag and can be set to f1 for full-sib families, hw for HWE model or ploidy for an assumption-free model. For the first two models, SUPERMASSA imposes some constraints given the expected individual genotype distribution by considering a cross of two heterozygous parents or HWE for natural populations, for example. For the last model, no constraints on the genotype distribution are imposed. By default, approximate inference based on a greedy maximum likelihood (ML) approach is performed. It is faster and expected to provide similar results as exact maximum *a posteriori* (MAP) inference in most cases. However, if one wants to use exact inference, the –exact or -e flag should be set. For details on these approaches, see [18, 21].

Besides the data to be analyzed, other expected values include a ploidy range (–ploidy_range or -M), e.g., 2:16 for searching all even ploidies from two through 16, and a sigma range (–sigma_range or -V), e.g., 0.01:1:0.05, with the lower bound, higher bound and the step values separated by : (colon). These ranges should be modified according to the species and genotyping technique being used. Another quality criterion that one may want to adopt is to establish a naive reporting posterior probability threshold (–naive_reporting or -n). The so-called naive reporting probabilities are attributed to each individual after classification with no consideration of any underlying genetic model. Very good genotype calls are expected to have a posterior probability of 0.90 or higher.

#### VCF2SM options

In addition to the required arguments for running SUPERMASSA, we included some options for quality filtering and to speed up the analysis process. First of all, one must choose which VCF field the read depths should be extracted from: AD (allelic depths for the reference and alternative alleles in the order listed), RA/AA (reference/alternative allele depths) or RO/AO (reference/alternative allele observation counts) are commonly found in VCF files produced by TASSEL-GBS, GATK or FREEBAYES, respectively. This information is usually found in the header of the VCF file and refers to the number of respective reference and alternative allele counts for each individual. Based on the expected ploidy level(s) for a given species, one may want to define both average minimum (–minimum_depth or -d) or maximum (–maximum_depth or -D) depths per sample per variant site (not including the parents for F_1_ families). From our experience, a minimum of 5, 15 and 25 reads on average should work well for di-, tetra- and hexaploid species, respectively. Each situation should be analyzed carefully, while taking into account the experimental protocol involving enzyme choice, number of runs and library plexing, for instance. Moreover, if duplications are expected in the species under consideration, one may restrict the genotype calls to loci with a certain maximum depth. For instance, duplicated loci along the genome might cause segregation distortion in full-sib families and complicate subsequent linkage analysis. Again, choosing a maximum depth should rely on the design of the GBS experiment and on the biological knowledge of the species.

When a species (or a particular polymorphism) has unknown ploidy level, one can infer it from a range given by the SUPERMASSA argument (–ploidy_range or -M), as indicated in the previous section. For selecting the best ploidy level, the software uses MAP probability among the tested ploidies. It has been noticed that it is good practice to define a threshold as high as 0.80 for the posterior (–post or -p) [9]. This is because very dispersed marker data can yield low posteriors for multiple ploidy levels which may lead to a compromised classification. For the tested range of ploidies, one can filter based on the most likely ploidy level given biological information, by using –ploidy_filter or -f. The proportion of missing data can be controlled by –callrate or -c, so that a locus will only be output if it reaches the specified threshold.

It is common in GBS sequencing runs to include samples from different projects. A user may therefore want to do quantitative genotyping separately for different subsets of the samples. When selecting the samples to be included, one can choose from either a sample pattern identifier (using –geno_pattern or -g) or, alternatively, a numerical range of individuals (–geno_range or -r). In a similar manner, one can specify all parent replicates with a pattern (–par1_pattern or -1 and –par2_pattern or -2) or ranges (–par1_range or -k and –par2_range or -l) for a full-sib family.

Finally, computational time for VCF2SM is reduced by using multithreading. For that, the flag –threads or -t indicates the number of threads to be used. We performed all the following analyses using Ubuntu 12.04 LTS as operating system in a cluster node with 16 cores in total (Dell R620) and 128 GB RAM. In fact, although the modified TASSEL-GBS uses more memory, we notice that 16 GB usually suffice for most applications.

### Examples from GBS data

We tested VCF2SM with publicly available GBS data from two autotetraploid species, potato (2*n*=4*x*=48) and alfalfa (2*n*=4*x*=32). In addition, we also studied a dataset from switchgrass (2*n*=4*x*=36), an outcrossing tetraploid species which behaves like a diploid. GBS experiments were performed in order to increase the read depths for two diverse panels with 84 potato cultivars [23] and 189 alfalfa accessions [28], with average read depths per individual of 70 × and 27 ×, respectively. On the other hand, GBS experiments for two F_1_ mapping populations with 389 alfalfa [25] and 129 switchgrass [26] full-sibs did not aim for higher read depths so that their averages were less than 1 × each. In the previous studies, although genotype calling for both diverse panels was achieved through allele dosage, only SDM from diploid-based genotype call software were used for linkage analyses in both full-sib populations.

#### Potato diversity panel data

For the potato panel, 135,193 loci in a VCF file were provided as supplementary material by the authors [23] and we used it directly with VCF2SM under the HWE model (-I hw). We specified the field to get allele depths from using -a RA/AA as the file was obtained by GATK. Here, we initially called the genotypes using their read counts by fixing a ploidy level of four (-M 4) or by varying it from four to six (-M 4:6) while only selecting tetraploid loci (-f 4). No other filtering criteria was used. The fixed ploidy level returned all 135,193 loci. We compared the genotypes called by SuperMASSA with the original calls obtained with FREEBAYES, to assess the agreement rate between these two strategies. Results showed that 94.3% of the genotype calls were identical, indicating that both methods agreed largely in differentiating allele dosages. Some differences were expected because the calling algorithms for SUPERMASSA and FREEBAYES differ in principles. When we allowed the ploidy level to vary between four or six, 70,343 tetraploid loci were returned, after excluding 64,850 (48%) loci classified as hexaploid. We observe this result when SUPERMASSA is confronted with data that is too scattered. Under these conditions SUPERMASSA has a tendency to classify some loci to the highest ploidy level provided in order to fit more classes of allele dosage. Most of the hexaploid loci present a low posterior probability after all, and we should not rely on this classification alone for selecting markers to be studied [9, 18].

We also considered further quality filtering criteria, such as a high posterior probability for the most likely ploidy (-p 0.80) and individual naive reporting probabilities (-n 0.90). The genotype call was also limited to an average minimum and maximum read depths of 15 and 500 per individual (thus -d 15 and -D 500), respectively. Still, even considering a high population call rate (-c 0.75), the analyses returned 96,078 or 52,093 tetraploid loci depending on whether the ploidy was fixed (-M 4) or not (-M 4:6). It is worth mentioning that the approach used in the original paper does not allow testing different ploidy levels simultaneously. In fact, the user has to provide a fixed ploidy level. However, for some species the ploidy level is unknown or varies. This new function allows one to test which ploidy better fits the data for each polymorphism, individually. Even if the ploidy level is known (as it is for potato), one can still try other ploidies as an additional filtering criterion. Here, we discarded those markers classified as hexaploid and continued the analysis with the markers classified as tetraploid only.

**Fig. 1:**
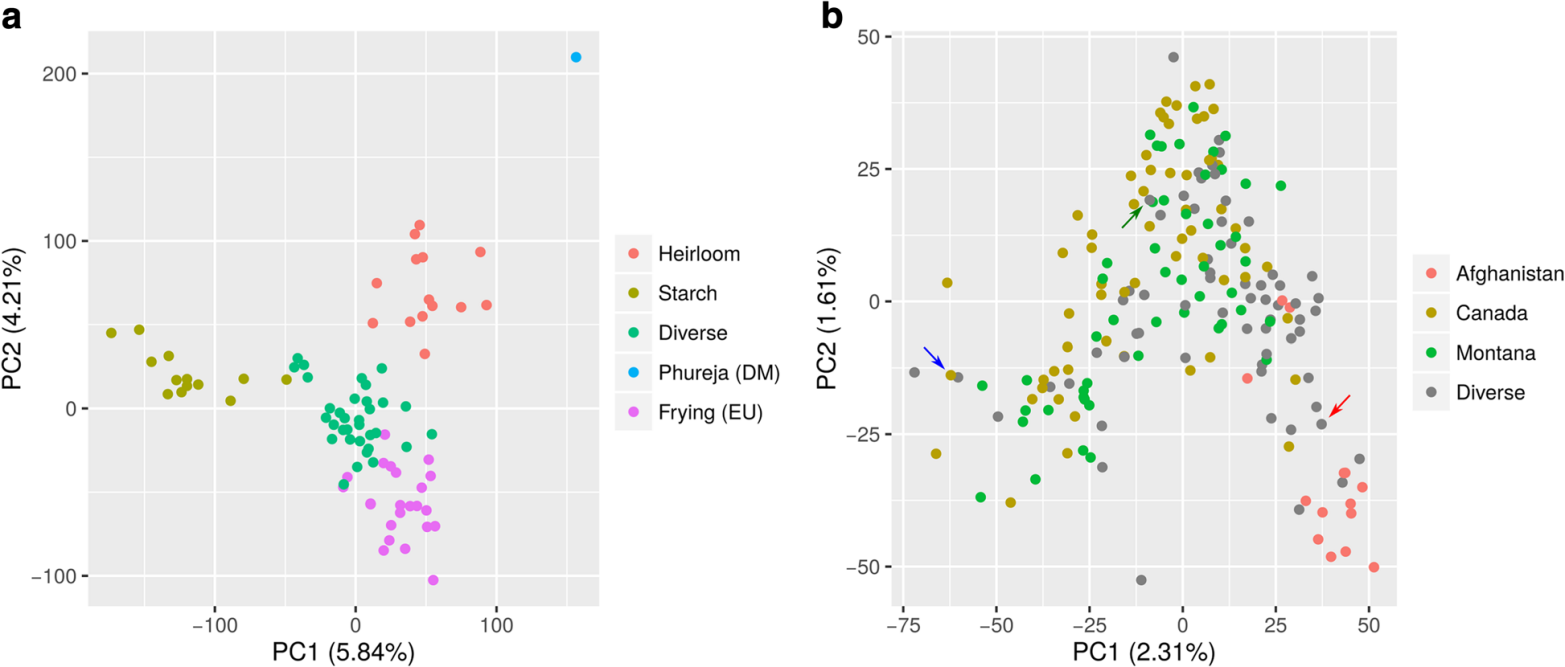
Principal component analyses (PCAs) for two diverse panels of autotetraploid species. We called genotypes using VCF2SM with ploidy level of four. PCA was carried out for 135,193 and 74,790 loci for diverse panels of 83 potato cultivars (**a**) and 189 alfalfa accessions (**b**), respectively. **a** There were four groups and an additional diploidized potato (‘Phureja’) previously identified [23]. **b** Only genotypes from Afghanistan were somehow grouped. Red, green and blue arrows indicate the same genotypes (‘wilson’, ‘saranac_G’ and ‘rambler’, respectively) highlighted in [24]

After the VCF production, we re-coded each genotype with integers from 0 (0/0/0/0) to 4 (1/1/1/1) according to the alternative allele dosage. Using the PCAMETHODS R package [29], we ran principal component analysis (PCA) for each set of markers [see Additional file 1: Figure S1]. We noticed that there was no evident discrepancy between the groups obtained using the 135,193 tetraploid loci classified here (Fig. 1a) and the ones obtained by the original paper [23]. The sums of the variance explained by the first two principal components (PCs) for each set of markers produced here differed slightly (from 10.04% to 12.06%). Some differences on the grouping pattern could be noticed when the filtered dataset was analyzed, particularly with regards to the second PC [see Additional file 1: Figure S1]. We observed almost identical results when using exact inference (-e) or the default approximation. We avoided the exact inference approach for the next datasets because it is extremely time-consuming and the benefits of using it are likely to be only minor for GBS-based techniques.

#### Alfalfa diversity panel data

For the alfalfa panel, we ran the modified TASSEL-GBS using raw sequence data for 189 individuals from NCBI (BioProject PRJNA287263 [28]). Out of 1,906,719 tags, 52.41% were aligned against the diploid relative *M. truncatula* L. genome [30] (Mt4.0v1 DOE-JGI, http://phytozome.jgi.doe.gov/) using BOWTIE 2 [31]. Finally, exact allele-specific depths were recorded in VCF files for 399,687 loci.

We ran VCF2SM under the HWE model (-I hw) with fixed (-M 4) or a range (-M 4:6) of ploidy levels for comparison. Initially, only the minimum and maximum average count filters were applied as -d 15 and -D 500, to avoid very low or very high read depths. In both cases, we just used the loci classified as ploidy level of four (-f 4). A total of 74,790 markers were kept in the first case. The second set of markers contained 17,268 loci because we excluded loci classified with a ploidy of six. As a result of further quality filtering criteria (-p 0.80, -n 0.90 and -c 0.75), the final numbers of loci retained became 50,929 and 11,690, respectively.

Using PCAMETHODS for running PCA for each set of markers, we noticed that the genotypes were similarly distributed along the two first PCs [see Additional file 1: Figure S2], regardless of the filtering criteria used. This high density genotyping approach often provides a certain amount of duplicates (redundant loci). We excluded these (around 26%) and individuals were distributed in the same way as before. The first two PCs accounted for 3.80% to 5.10% [see Additional file 1: Figure S2] of the total variance. Apart from the genotypes from Afghanistan, the remaining accessions did not show any other clear clustering (Fig. 1b), as observed previously [24].

To compare the results obtained via VCF2SM with alternative genotyping methods, we reanalyzed the raw sequencing data from [28] using FREEBAYES, which is also appropriate for diversity panel datasets. We initially aligned the deconvoluted raw sequencing reads against the *M. truncatula* genome, using BOWTIE 2 [31] with the –very-sensitive-local argument. Next we ran FREEBAYES with a fixed ploidy of four, requiring at least five reads of the alternative allele, a minimum read mapping quality of 1 and a minimum base quality of 5. Variants were then filtered to remove non-biallelic or monomorphic sites, with an assigned quality score lower than 20 or more than 50% missing data, as well as sites with less than 15 or more than 500 read counts on average.

This strategy yielded 27,076 variants, close to the number obtained by [28] (26,163). We then applied VCF2SM on this data set using the same four scenarios described above: a fixed ploidy of four, with no additional filters or more stringent criteria (-p 0.80, -n 0.90 and -c 0.75), and ploidy levels of four and six, with or without these additional filters. When using the most permissive setting, all variants were retained and the genotyping identity between the two methods was 93.69%. Using more stringent filters reduced the number of sites to 21,382, but increased concordance to 98.01%. Alternatively, filtering out loci with an estimated ploidy of six and applying more stringent quality criteria reduced the number of variants to 10,083 and 8,332, again increasing the genotype agreement rate to 96.51% and 98.32%, respectively.

Because this data set contains individuals from a diversity panel, it is expected that many polymorphic sites show low frequency of the alternative allele. In this situation, the majority of individuals are likely to be homozygous for the reference allele, which in turn simplifies genotype calling. Interestingly, when we compared genotype calls only for heterozygotes, the agreement rate between the two methods dropped to 79.28% in the less stringent scenario. Adding more stringent filters increased this rate to 87.82% and, lastly, filtering out loci with an estimated ploidy of six resulted in 90.20% of matching calls. Hence we note that the additional filters provided by VCF2SM allowed the exclusion of less reliable genotype calls, which had passed the standard filters applied to the FREEBAYES results.

Although we used very stringent criteria for VCF2SM parameters with the TASSEL-GBS pipeline, our method obtained a higher number of classified markers compared to [28] using GATK and FREEBAYES. As a probabilistic model, the SUPERMASSA algorithm allows filtering genotypes according to their probability of being in a class given the data. This can still be informative even if there is no genetic model underlying the analyzed population, as it uses the allele ratio to inform on the more likely genotypes.

#### Alfalfa F_1_ population data

For the alfalfa full-sib family, we ran the modified TASSEL-GBS using raw sequence data from 389 individuals (BioProject PRJNA245889 [25]) as done previously with the diverse panel. Out of 3,889,791 tags, 57.15% were aligned against the *M. truncatula* genome. Twelve replicates for the parents ‘DM3’ and ‘DM5’ each were available and used as a relevant input for adding more constraints to the SUPERMASSA F_1_ model (-I f1). A total of 474,327 loci were recorded in VCF files.

Following the same strategy for comparison, we ran all the markers in VCF2SM with no filtering criteria other than the ploidy level (-f 4) and minimum and maximum average depths (-d 15, -D 500). The fixed ploidy level of four (-M 4) resulted in 59,480 loci, while when the hexaploid level was also tested (-M 4:6), 20,396 tetraploid loci were kept. However, when additional filtering criteria were applied (-p 0.80, -n 0.90 and -c 0.75), only 230 and 80 loci remained. This is probably due to the non-optimized protocol for increasing the read depths. We therefore relaxed the naive reporting probabilities by letting all individuals to keep their assigned genotypes (-n 0.00) and a total of 58,375 and 19,837 loci were obtained.

To be more conservative, we used the 19,837 marker dataset for further analysis. The genotypes were re-coded from 0 through 4. We also filtered out 5,803 either monomorphic or redundant loci, which are non-informative in linkage analysis. According to the type of cross of the remaining 14,034 markers, there were 9,989 SDMs resulting from nulliplex-simplex or simplex-simplex crosses, and 4,859 MDMs as a result of higher dosage crosses. It is important to mention that these MDMs do not only represent more than one third of the loci spanning the genome, but also that they are more informative than SDMs for linkage mapping analysis. Notice that, while keeping missing data ≤ 25%, we increased the number of markers in comparison to the previous study, which analyzed 8,527 markers with ≤ 50% missing genotype calls [25].

**Fig. 2:**
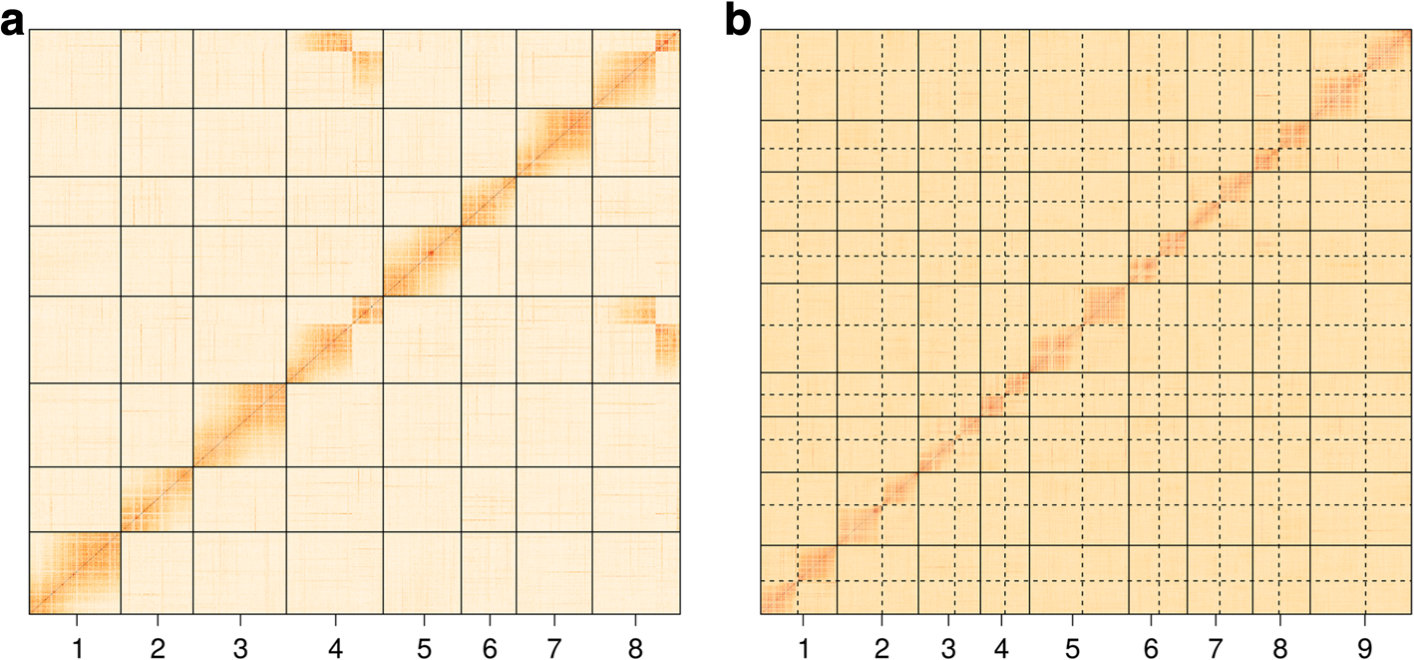
Heatmaps of absolute pairwise correlations between markers from two mapping populations. In the heatmaps, the darker the color, the higher is the correlation between markers. Populations were composed by 389 alfalfa (**a**) and 129 switchgrass (**b**) full-sibs. Both species are tetraploids, but switchgrass has been thoroughly diploidized. We classified the markers under a range of ploidy levels (from four to six for alfalfa and from two to four for switchgrass) and selected for the lowest ploidy level (four and two, respectively). See text for additional parameters. Monomorphic and redundant markers were filtered out. Single dosage markers were also excluded to abbreviate the calculations. **a***Medicago sativa* is composed by eight chromosomes, as is the *M. truncatula* reference genome, here represented by 7,937 markers. Note a major translocation between chromosomes 4 and 8. **b***Panicum virgatum* genome has two sets of nine homoeologous chromosomes each (the pairs are separated by dashed lines). All chromosomes were represented in the heatmap by 16,263 markers

For characterizing the linkage disequilibrium generated by linkage in this mapping population, we simply calculated the pairwise marker correlation, by using the WGCNA R package [32] for dealing with big matrices. Then, we plotted heatmaps with the absolute correlation values between markers with more than two genotypic classes (Fig. 2a). All eight diploid chromosomes of the relative *M. truncatula* are represented by 7937 more informative loci (all except nulliplex-simplex crosses), with the number of markers ranging from 211 (chromosome 6) to 1147 (chromosome 4). A translocation between chromosomes 4 and 8 is evident as previously reported [25]. The same grouping pattern was observed under other filtering criteria, although increasing the number of markers reduced the correlations [see Additional file 1: Figure S3]. Previously, the linkage maps were presented as two parental maps with 32 linkage groups (LGs) each and 3591 SDMs in total. Notice that, although we have failed in using naive reporting probabilities for filtering purposes, the genotype calls provided here were good enough to reveal the linkage disequilibrium structure along the genome. A GBS experiment properly optimized for increasing read depths would allow the use of the naive reporting probabilities because improved dosage class assignments are expected.

#### Switchgrass F_1_ population data

We also ran the modified TASSEL-GBS using raw sequence data for 129 full-sibs of switchgrass and their parents ‘U518’ and ‘U418’ from NCBI (BioProject PRJNA201059 [26]). Out of 3,203,382 tags, 93.21% were aligned against the *P. virgatum* genome [30] (v3.1, DOE-JGI, http://phytozome.jgi.doe.gov/) using BOWTIE 2 [31]. Finally, exact allele-specific depths for 5,356,352 loci were recorded in VCF files. This amount includes all putative polymorphic markers from the whole dataset, which is composed by an additional half-sib population of 168 individuals and a diverse panel of 540 individuals from 66 populations.

Besides testing the genotype call under the ploidy level of two (-M 2), we also searched ploidy levels ranging from two to four (-M 2:4) and from two to six (-M 2:6). Because switchgrass is a tetraploid species thoroughly diploidized, in the first two cases, only diploid genotypes were kept (-f 2), while in the last case, both diploid and eventual tetraploid genotypes were retained (-f 2:4). With no filtering criteria other than the minimum and maximum average read depth (-d 3, -D 300), we ended up with 498,310, 79,383 and 111,551 markers, respectively. Once additional criteria were used (-p 0.80, -c 0.75), these numbers became 474,252, 74,504 and 98,409. Notice that we did not filter for the naive reporting probability, because this yielded very few markers.

Taking the 74,504 more stringently filtered markers, we re-coded the genotypes as 0 (0/0), 1 (0/1) and 2 (1/1) for further analysis. After excluding 23,879 monomorphic or redundant markers, 34,361 and 16,264 markers were segregating in 1:1 and 1:2:1 ratios, respectively. We computed the pairwise correlations between the most informative markers (1:2:1) using WGCNA, and a heatmap showed 18 LGs as expected from the reference genome (Fig. 2b). The set of 474,252 markers resulted in 16,264 markers segregating 1:2:1 and showed similar grouping pattern. From the set of 98,409 markers, there were 74,498 classified as diploid (mostly the same ones from the -M 2:4 search) and 23,911 classified as tetraploid. The same pattern of 18 LGs was observed with the 16,271 most informative diploid markers. The re-codification of the remaining 13,209 tetraploid MDMs included genotypes from 0 to 4, but no grouping pattern was evident [see Additional file 1: Figure S4]. Altogether, switchgrass appears to be entirely diploidized and additional tetraploid classification proved to be merely artifactual due to lack of quality control of the genotype calls.

Finally, we converted the respective 0, 1 and 2 codes to *a*, *ab* and *b*, following [12]’s notation as required by ONEMAP (developing version, https://github.com/augusto-garcia/onemap), that is an R package for building linkage maps. A very conservative chi-squared test (*p*<0.10) was carried out on the 50,625 polymorphic markers, which excluded 39,317 distorted markers. Trying to build a *de novo* genetic map, we used log of the odds (LOD) score > 12 and recombination fraction ≤ 0.35 for grouping the 11,308 remaining markers. A total of 6,555 (58.0%) markers were grouped in 18 major LGs with the number of markers ranging from 200 (LG 11) to 754 (LG 18). In addition, there were five intermediate size groups (from 15 to 59 markers), 600 very small groups (from two to eight markers each) and 3187 unlinked markers. Interestingly, 860 (13.1%) markers were allocated in a different LG from the expected chromosome. These disagreements may be related with translocations, reference genome misassembly or genotyping errors. Despite having ordered markers by the reference genome, we found it very difficult to estimate a final map. This is likely related to the non-filtered genotype calls (-n 0.00), which carry a lot of miscalled genotypes with serious implications for correct multipoint genetic distance calculations. Therefore, optimized GBS pipelines for increasing the number of reads is mandatory to achieve more accurate genotype calls.

### Computational requirements

The most computational demanding step of the complete pipeline is initial SNP calling, regardless of whether it is carried out with TASSEL-GBS, FREEBAYES, GATK, or other methods. Once the allele depth counts have been obtained, running VCF2SM requires relatively little resources. For instance, analyzing the 27,076 loci of the alfalfa diversity panel with a fixed ploidy level took approximately 13 min, when using 16 parallel threads. Fitting both the ploidies of four and six increased the runtime to 17 min. As another example, analysis of the 59,480 variants of the alfalfa F_1_ progeny with a single ploidy level took required 50 min, because the number of samples is larger. Testing two ploidy levels took roughly 80 min. Fitting more ploidy levels increases runtime, but only a few levels usually need to be tested for the majority of species with known ploidy.

Memory requirement is also low and VCF2SM can be run in personal desktop computers. Analysis of the 189 individuals of the alfalfa panel, in 16 threads, required roughly 1 GB of RAM. More concurrent threads require more memory, but the trade-off between runtime and memory can easily be adjusted to match the resources available to the researcher.

## Conclusions

In the current literature, we have noticed that the application of GBS-based technologies in polyploids is limited by the use of diploid-like genotype calls. This is likely because there were no bespoke bioinformatic pipelines with the ability to enable polyploid based quantitative genotyping. This limited previous studies from pursuing higher read depths (e.g., [25]). VCF2SM provides a simple and useful integration between VCF files and SUPERMASSA software for dosage genotype calling. VCF files can be obtained by using TASSEL-GBS modified for storing true read depths from GBS experiments.

Read depths for each variant allele were used in SUPERMASSA to estimate the allele dosage in two autotetraploid species, potato and alfalfa. We showed that the outputs are suitable for population and linkage genetics analyses and the results highly agreed with those previously obtained [23, 25, 28]. For switchgrass, a diploid-like outcrossing species, linkage was indicated from the markers we obtained [26]. Our approach shows that users will get results comparable to or better than those from existing tools for fixed ploidy levels.

In fact, other genotype calling packages for polyploids, such as FREEBAYES and FITTETRA, are intended only for species with known ploidy level, limiting their usage over a more general polyploid framework, such as some with higher, mixed or unknown ploidy levels. Namely, FITTETRA is limited to tetraploid species. Moreover, these programs do not consider important genetic information underlying the distribution of genotype classes in F_1_ populations or in diversity panels, whereas SUPERMASSA does. This is specially important for providing additional constraints on the genotype calling process, because SUPERMASSA uses the genotype distribution a priori in the inference procedure. Implementing a genetic model underlying allele and genotype class frequencies could also prove useful in the genotype calling procedures for outcrossing diploid species. Finally, we showed that testing a range of ploidies and keeping only loci that match the expected level for a given species provides an important quality filtering criterion.

VCF2SM was first intended for polyploid species, but it can be used for hybrids or outcrossing diploid species if researchers wish to get genotype calls based on the models implemented in SUPERMASSA. Thus, these species can potentially benefit from this integration. However, this approach should be used with caution, because the interpretation of higher ploidy levels for a locus may be related with not fully diploidized regions, polysomy or even structural variations, such as copy number variations (CNVs), rather than the genome ploidy level itself.

The difficulty of determining the allele dosage has been pointed out as a likely limitation for genetic studies in polyploid species. Although most of the development in methods and tools for studying these species relate to autotetraploids, we believe that proper models can take advantage of the dosage information for increasing prediction accuracies in genome-based selection [33], genetic mapping [34], performing genome-wide association studies [35] and depicting relationship among individuals in population studies [36] for other autopolyploid species. VCF2SM thus provides the first solution for getting genotype information for species with almost any even ploidy level from GBS through SUPERMASSA models.

Partially due to the lack of methods and tools for dealing with MDMs, they have been discarded in autopolyploid mapping studies under the reasoning that SDMs would primarily represent the genome of these species. Our analyses have shown that this might not be true given the datasets analyzed here. This is in agreement with the findings of [9] for sugarcane. Using GBS data from full-sib populations, we demonstrated the potential of our method in calling genotypes for studying linkage mapping independently of the ploidy level of the species. For the diploid-like species, genotype calls were useful for grouping but not for estimating map distances. Importantly, GBS protocols need to be optimized for increasing the read count so that genotypes can be called more accurately.

## Availability and requirements

- Project name: VCF2SM
- Project home page: https://github.com/gramarga/vcf2sm
- Operating systems: any supporting Python 2.7 (tested on Linux)
- Programming languages: Python 2.7
- Other requirements: SuperMASSA [21] source code available at https://bitbucket.org/orserang/supermassa
- License: GNU GPL
- Any restrictions to use by non-academics: license needed

## Additional file

Additional file 1 Supplemental figures from analyses with different sets of markers. (PDF 2515 kb)

## Abbreviations

CNV: Copy number variation
GBS: Genotyping-by-sequencing
indel: Insertion-deletion
LG: Linkage group
MDM: Multiple dosage marker
NGS: Next generation sequencing
PCA: Principal component analysis
SDM: Single dosage marker
SNP: Single-nucleotide polymorphism
VCF: Variant call format
