# Supplementary material for: A fully automated pipeline for quantitative genotype calling from next generation sequencing data in autopolyploids
Source: BMC Bioinformatics. 2018 Nov 1;19:398. doi: 10.1186/s12859-018-2433-6 (PMC6211426; doi:10.1186/s12859-018-2433-6)
Supplement: Supplementary file 1 — Supplemental figures from analyses with different sets of markers. (PDF 2515 kb) [file 12859_2018_2433_MOESM1_ESM.pdf]

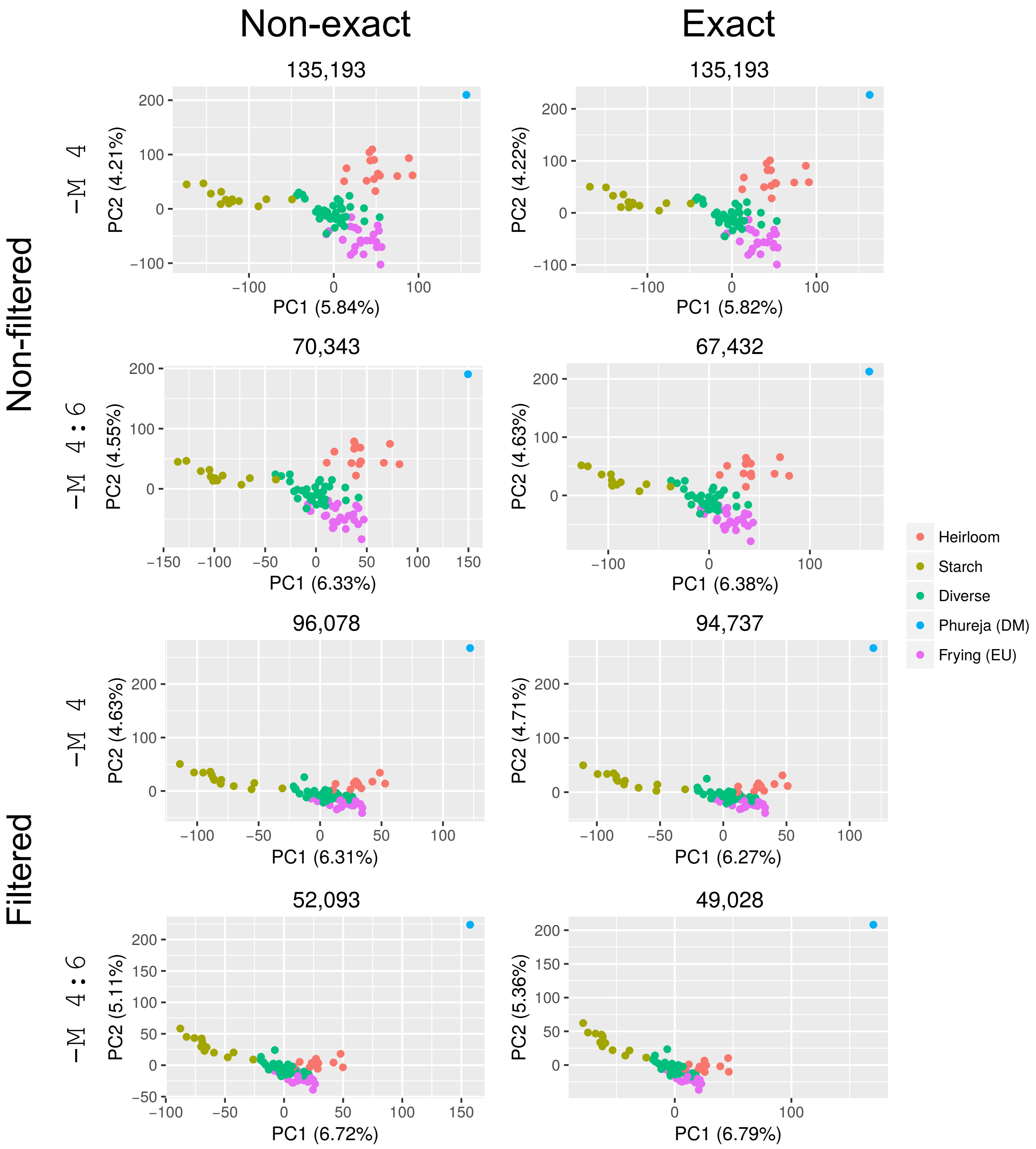

Figure 1 Principal component analyses (PCAs) for a diverse panel of 83 autotetraploid potato cultivars. Read depth information from 135,193 markers was analyzed using VCF2SM under different parameters. Each plot axis represents the first two principal components (PC) and their respective amount of variation explained (within parenthesis). Number of analyzed markers (above each plot) varies according to the type of inference (exact or non-exact), the use or not of filtering criteria (`-p 0.80`, `-n 0.90`, `-c 0.75`) and the tested ploidy levels (`-M 4` or `-M 4:6`). See text for details. Differences between the distribution of individuals were found basically among plots with filtered and non-filtered markers.

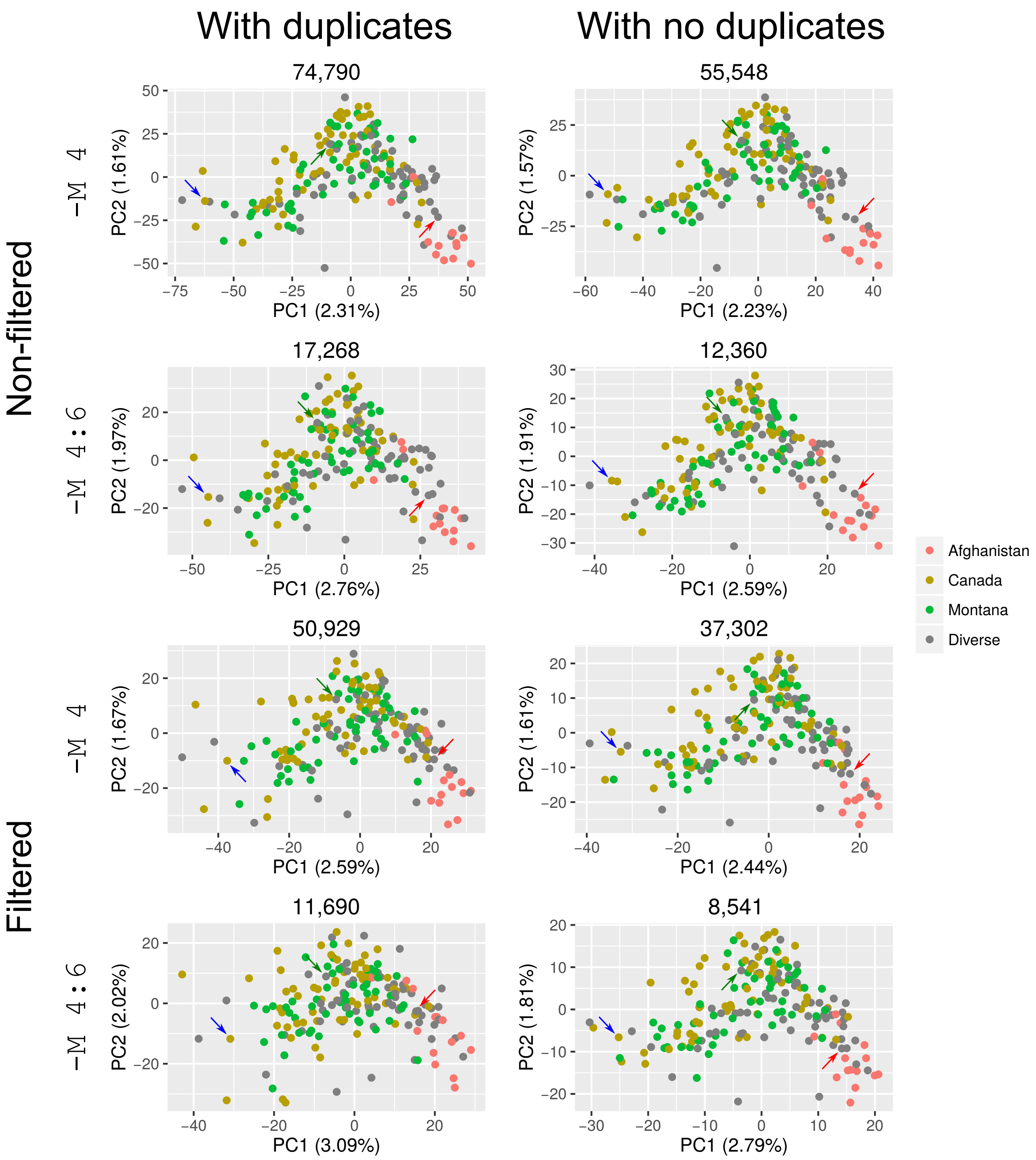

Figure 2 Principal component analyses (PCAs) for a diverse panel of 189 autotetraploid alfalfa accessions. Read depth information from ∼400k markers was analyzed using VCF2SM under different parameters for comparison. Each plot axis represents the first two principal components (PC) and their respective amount of variation explained (within parenthesis). Number of analyzed markers (above each plot) varies according to the presence or not of duplicate markers, the use or not of filtering criteria (`-p 0.80`, `-n 0.90`, `-c 0.75`) and searched ploidy levels (`-M 4` or `-M 4:6`). See text for details. There were no important differences between the distribution of individuals among plots. Red, green and blue arrows indicate the same genotypes ('wilson', 'saranac_G' and 'rambler', respectively) highlighted in [23]

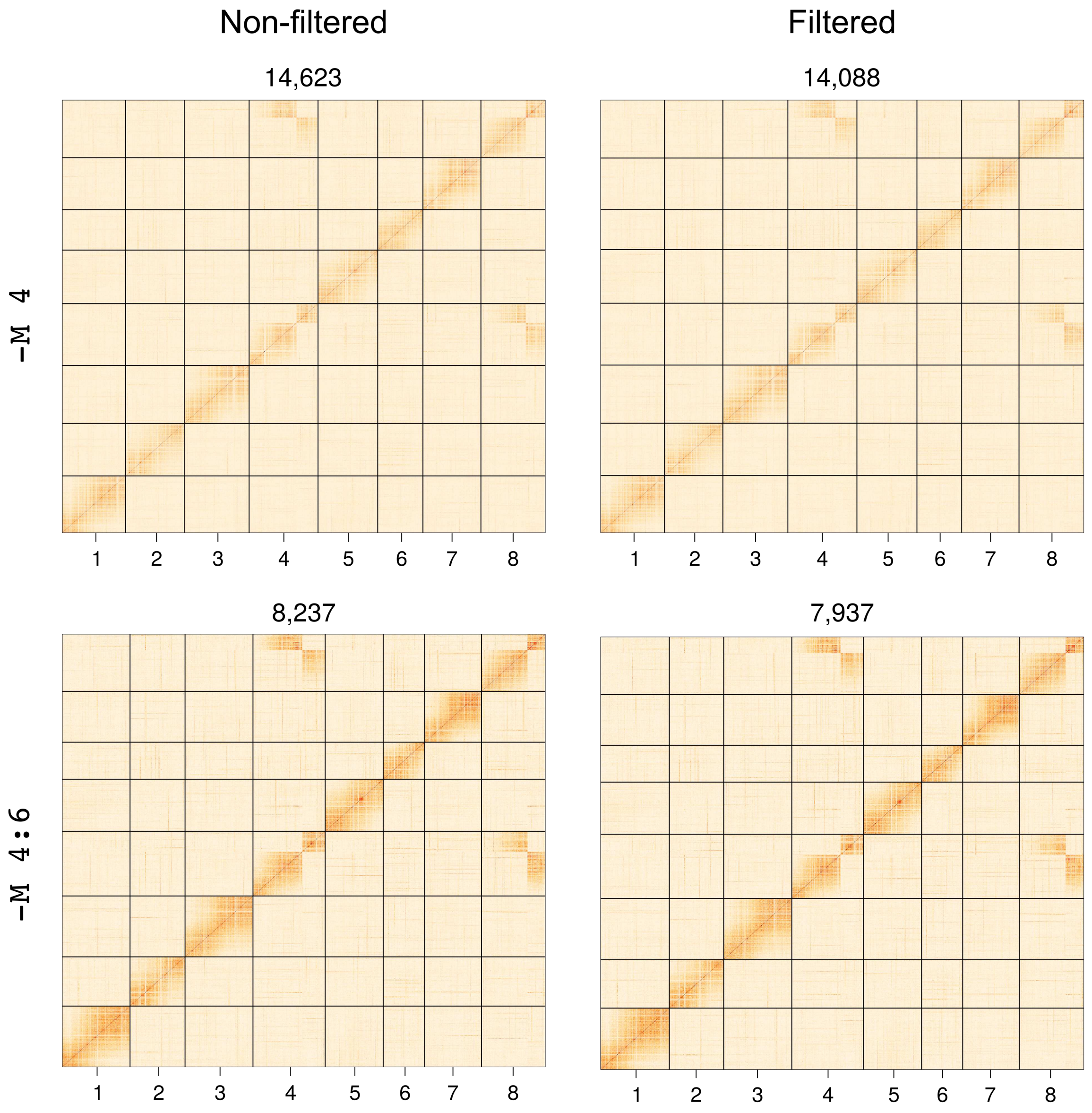

Figure 3 Heatmaps of absolute pairwise correlations between markers from a mapping population of 389 alfalfa full-sibs. In the heatmaps, the darker the color, the higher is the correlation between markers. We selected only tetraploid markers after testing ploidy levels of four (`-M 4`) or from four to six (`-M 4:6`). Number of markers (above each plot) changed slightly when some filtering was applied (`-p 0.80`, `-c 0.75`). Monomorphic and redundant markers were filtered out. Single dosage markers were also excluded to abbreviate the calculations. Filtering out markers classified with a ploidy level of six seemed to work better than including additional filtering criteria alone. All the eight chromosomes were represented here. A major translocation between chromosomes 4 and 8 is still evident.

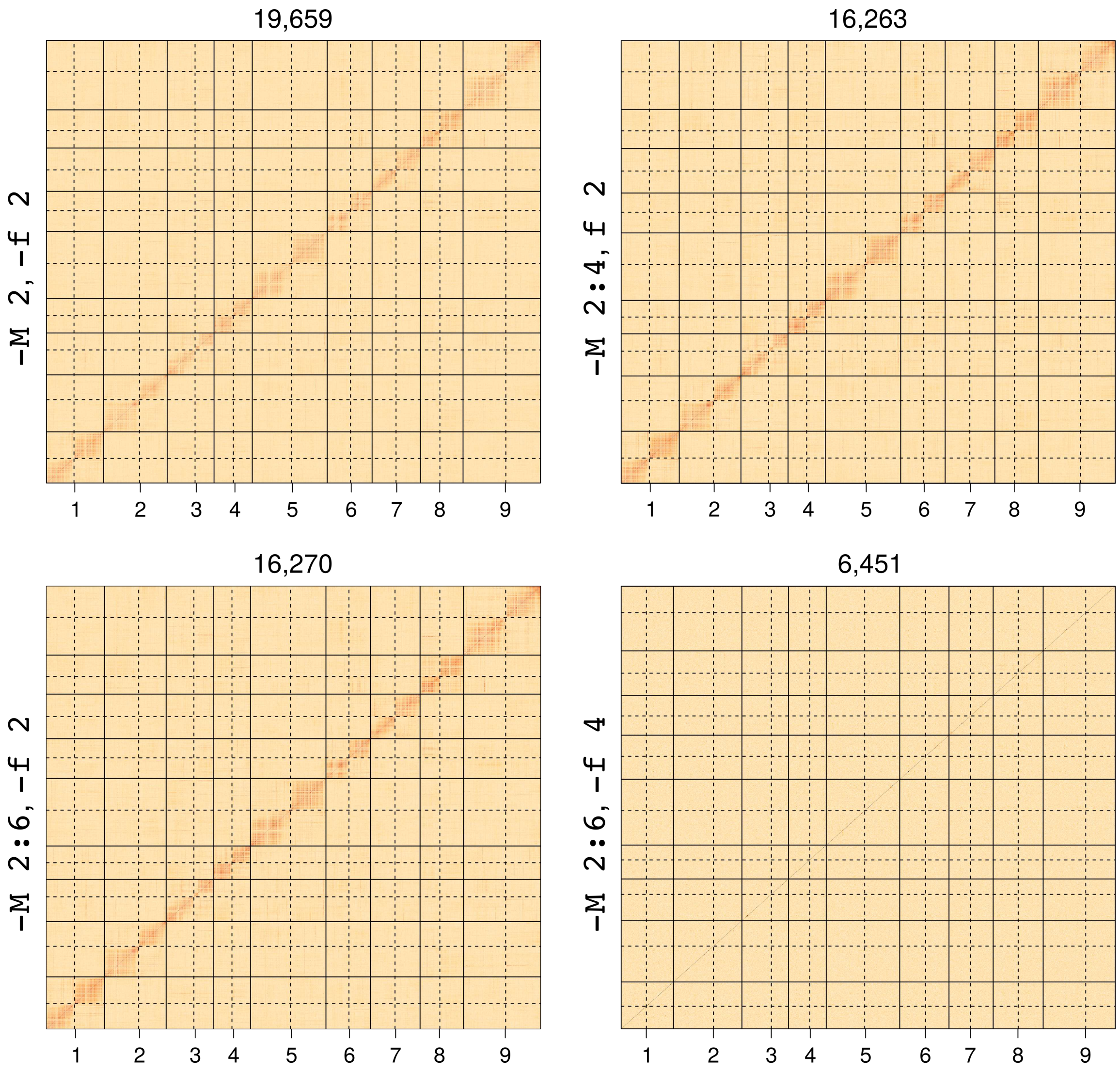

Figure 4 Heatmaps of absolute pairwise correlations between markers from a mapping population of 129 switchgrass full-sibs. In the heatmaps, the darker the color, the higher is the correlation between markers. We selected only diploid markers (`-f 2`) after testing ploidy levels of two (`-M 2`), from two to four (`-M 2:4`) or from two to six (`-M 2:6`). In this last case, tetraploid markers (`-f 4`) were also selected. Some filtering criteria (`-p 0.80`, `-c 0.75`) were used. Monomorphic and redundant markers were filtered out. Markers segregating 1:1 were also excluded to abbreviate the calculations. The species behaves indeed like a diploid, because no evidence of linkage disequilibrium was found among tetraploid markers. All the nine homeologous chromosome pairs (separated by dashed lines) were represented here by the number of markers above each plot.
